# Integrating Single-Walled Carbon Nanotubes into Supramolecular Assemblies: From Basic Interactions to Emerging Applications

**DOI:** 10.1021/acsnano.4c06843

**Published:** 2024-10-21

**Authors:** Verena Wulf, Gili Bisker

**Affiliations:** † Department of Biomedical Engineering, Faculty of Engineering, Tel Aviv University, Tel Aviv 6997801, Israel; ‡ Center for Physics and Chemistry of Living Systems, Tel Aviv University, Tel Aviv 6997801, Israel; § Center for Nanoscience and Nanotechnology, Tel Aviv University, Tel Aviv 6997801, Israel; ∥ Center for Light-Matter Interaction, Tel Aviv University, Tel Aviv 6997801, Israel

**Keywords:** carbon nanomaterials, hydrogels, amino acids, peptides, DNA origami, micelles, fluorescence sensing, low molecular weight gelators

## Abstract

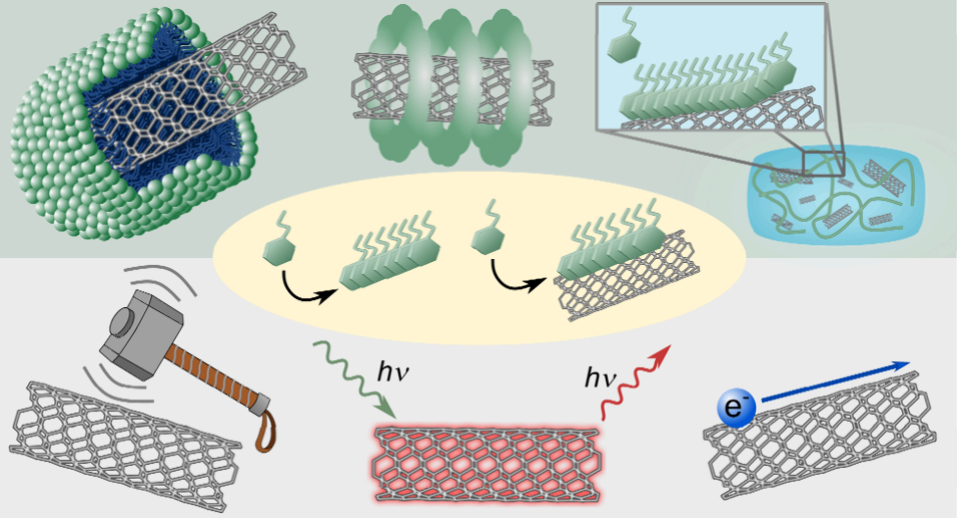

Integrating single-walled carbon nanotubes (SWCNTs) into supramolecular self-assemblies harnesses the distinctive mechanical, optical, and electronic properties of the nanoparticles alongside the structural and chemical properties of the assemblies. Organic molecules capable of forming supramolecular assemblies through hydrophobic, van der Waals, and π–π interactions have been demonstrated to be particularly effective in dispersing and functionalizing SWCNTs, as these same interactions facilitate the binding to the hydrophobic graphene-like surface of the SWCNTs. This review discusses a variety of self-assembling structures that were shown to integrate SWCNTs, ranging from simple micelles and ring structures to complex DNA origami and three-dimensional hydrogels formed by low-molecular-weight gelators. We explore the integration of SWCNTs into various supramolecular assemblies and highlight emerging applications of these composite materials, such as the mechanical enforcement of self-assembling hydrogels and leveraging the near-infrared (NIR) fluorescence properties of SWCNTs for monitoring the molecular self-assembly process. Notably, the distinctive NIR fluorescence of SWCNTs, which overlaps with the biological transparency window, offers significant opportunities for noninvasive sensing applications within the supramolecular platforms. Future research into a deeper understanding of the interactions between SWCNTs and different supramolecular frameworks will expand the potential applications of SWCNT-integrated supramolecular assemblies in fields like biomedical engineering, electronic devices, and environmental sensing.

## 1 Introduction

The integration of nanomaterials, including carbon-based nanomaterials, as well as plasmonic or magnetic metal nanoparticles, into organic supramolecular assemblies ranging from micelles, amino acid- or peptide-assemblies to supramolecular hydrogels, facilitates the development of innovative materials. These composite materials combine the distinctive properties of their constituents, such as, for example, the optical or electronic characteristics of the nanomaterial and the chemical or mechanical features of the incorporating supramolecular assemblies, leading to enhanced functionalities and emerging applications.

Among these advanced nanomaterials, single-walled carbon nanotubes (SWCNTs) stand out due to their distinctive structural and functional attributes, and their broad application spectrum. SWCNTs are tubular nanomaterials with a graphene-like carbon sp^2^-lattice. The structure of a SWCNT is described by the chiral vector (*n,m*), where *n* and *m* correspond to multiples of the graphene unit cells. The properties and, thus, the application of the SWCNTs are defined by their structure.^1^ Specifically, if the difference (*n–m*) is a multiple of 3, the SWCNTs are metallic, whereas otherwise, they are semiconducting with bandgaps inversely proportional to their diameter.^2,3^ With diameters ranging from <1 nm up to ∼2 nm and lengths varying from a few tens of nanometers to several micrometers,^4^ SWCNTs can effectively be considered as one-dimensional material. SWCNTs exhibit distinctive mechanical properties, including high elasticity, tensile strength, elastic modulus, and flexibility, especially compared to multiwalled carbon nanotubes.^5,6^ Further, SWCNTs are known for their thermal conductivity allowing them to withstand high temperatures, and they are applied in nanoelectronics due to their high electrical conductivity.^5^ Moreover, semiconducting SWCNTs display distinctive optical properties. Their bandgap structure allows for fluorescence excitation within the visible and near-infrared (NIR) wavelength range (500–900 nm) and fluorescence emission in the NIR region (900–1400 nm).^3,7^

The properties of the SWCNTs can be exploited in a plethora of applications, whether utilized as suspended SWCNTs^8,9^ or integrated into diverse material platforms, such as supramolecular assemblies and hydrogels.^10−16^ Notably, their fluorescence emission in the biological transparency window of tissues renders SWCNTs invaluable as fluorescence sensors for imaging and sensing in biomedical applications.^17−27^ Additionally, their electronic properties can be used in nanoelectronic devices,^28−31^ and their mechanical properties are exploited to reinforce hydrogels and other functional materials, enhancing their structural integrity and functionality.^32,33^

Due to their molecular composition, SWCNTs are highly hydrophobic. However, many applications require them to be suspended in aqueous solutions.^34^ One method to suspend SWCNTs is by their oxidation, leading to covalent functionalization of the graphene lattice with oxygen-containing groups, such as carboxyl- or hydroxyl-groups, enhancing SWCNT hydrophilicity. Yet, these covalent functionalizations of SWCNTs break the SWCNTs’ sp^2^-lattice, modulating the SWCNT properties, which may lead, for example, to a loss of fluorescence and electronic conductivity.^35^ To maintain these properties, SWCNTs are mainly suspended noncovalently by agents that can attach to the hydrophobic SWCNT surface and provide sufficient hydrophilicity to support stable SWCNT suspensions in water.^36^ These agents include, among others, DNA,^37−39^ surfactants,^40^ (amphiphilic) polymers,^41,42^ amino acids and their derivatives,^43,44^ peptides,^12,45,46^ and proteins.^36,47^

Interestingly, many of these agents used for suspending SWCNTs can form supramolecular assemblies between themselves, in the absence of SWCNTs. Such assemblies arise due to the inherent structural properties of these agents facilitated by van der Waals interactions, π–π stacking, and hydrogen bonding. Common examples include micellar assemblies of surfactants, lipids, and amphiphilic polymers, as well as tubular or fibrillary assemblies composed of amino acids, peptides, and proteins.^12,42,48,49^

**Scheme 1:**
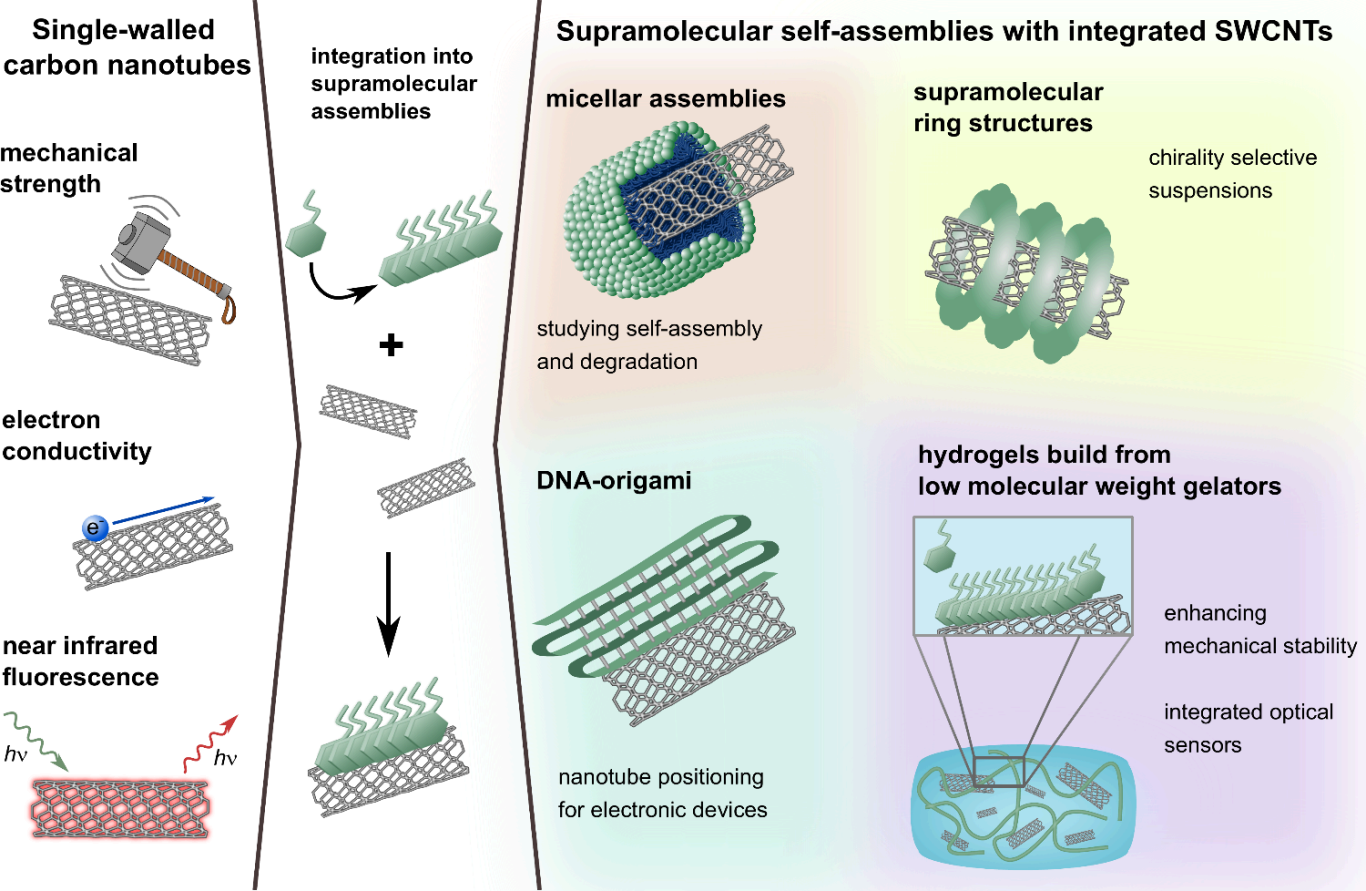
Single-Walled Carbon Nanotubes Integrated into Supramolecular Assemblies and Possible Applications

In this review, we explore the dispersion and integration of SWCNTs within various supramolecular assemblies, ranging from simple micellar structures of surfactants to more controlled assemblies like DNA-origami. Further, we discuss the incorporation of SWCNTs into three-dimensional supramolecular hydrogel structures formed by the self-assembly of low molecular weight gelators, mainly amino acids and peptides, that hold significant potential for biomedical applications. Focusing on noncovalently functionalized SWCNTs, which retain the physical and chemical properties of the SWCNTs, we elaborate on how supramolecular assemblies are utilized to selectively suspend SWCNTs and how SWCNTs enhance the properties or assist in the characterization of the supramolecular assemblies. Notably, there is particular interest in the application of SWCNTs integrated into supramolecular assemblies as optical NIR sensors reporting on the formation and degradation of the self-assemblies. While SWCNTs and their noncovalently attached coronae can be considered as supramolecular assemblies themselves, we will emphasize compounds that inherently form such supramolecular assemblies independently of SWCNTs, owing to their structural and chemical properties. While there are many interesting examples of SWCNT integration into supramolecular assemblies, many studies focus on the interaction of SWCNTs with these supramolecular assemblies, which underscores that applications in this area are still emerging, highlighting promising avenues for future research and development (Scheme 1).

## 2 SWCNTs in Micellar Assemblies

**Figure 1:**
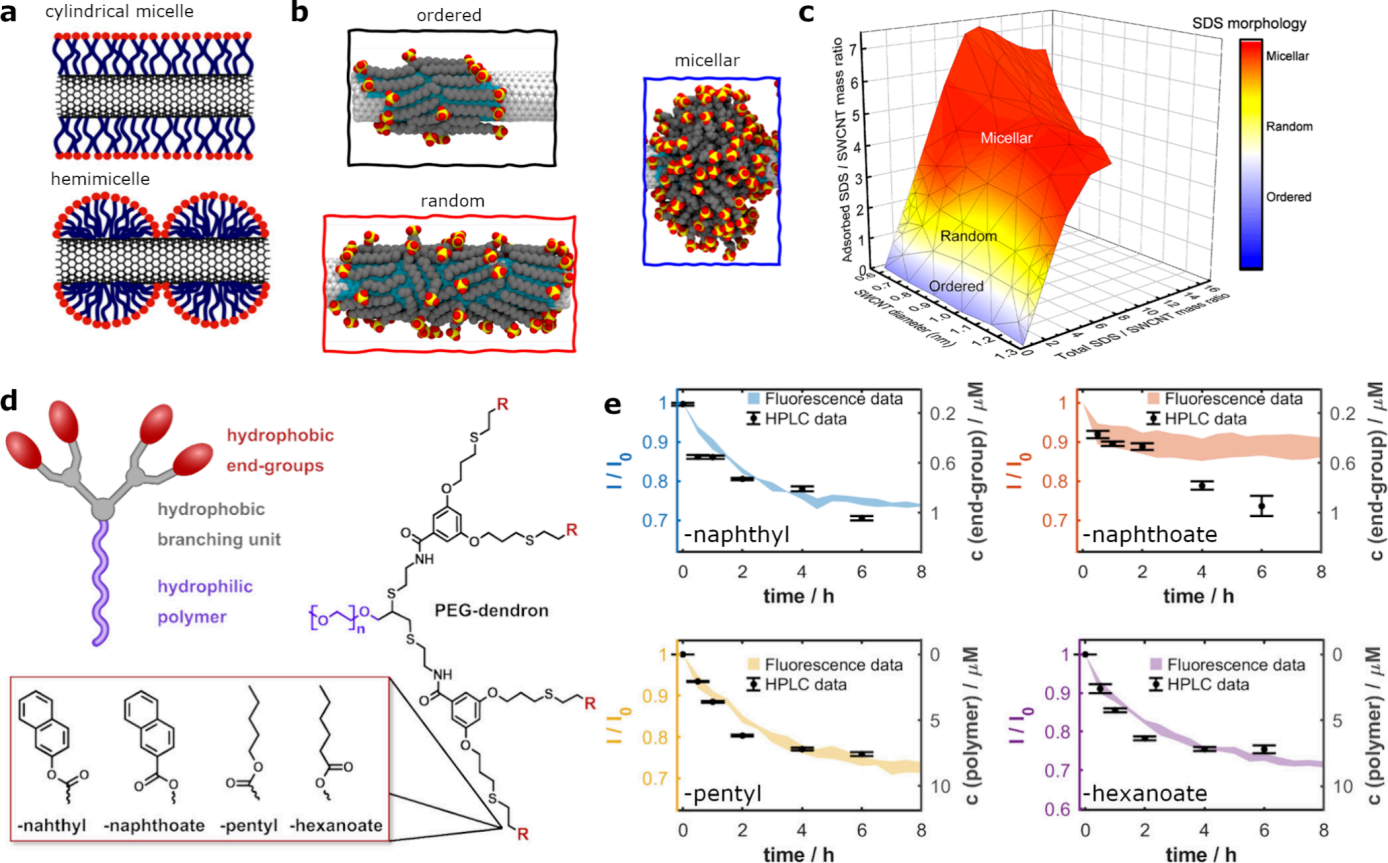
Formation and fluorescence measurement of micellar assemblies on the SWCNT surface. a) Schematic representation of possible micellar assemblies of surfactants around the SWCNTs. Reprinted with permission from ref (54). Copyright 2008 IOP Publishing. b) Concentration-dependent assembly of SDS on the SWCNT surface. c) Phase diagram of the different morphologies of SDS depending on the SDS/SWCNT mass ratio and the SWCNT diameter. Reprinted with permission from ref (49). Copyright 2024 American Chemical Society. d) Structure of polymer–dendron hybrids consisting of a hydrophilic PEG-polymer, a hydrophobic dendritic branching unit, and four hydrophobic (aromatic and aliphatic) end-groups connected via enzymatically cleavable ester bonds in different configurations. e) SWCNT fluorescence intensity changes (colored lines) and polymer degradation of the PEG-dendrons followed via HPLC measurements (black data points) after esterase enzyme addition for different PEG-dendrons, -naphthyl (blue), -naphthoate (red), -pentyl (yellow), and -hexanoate (purple). Reprinted with permission from ref (42). Copyright 2021 American Chemical Society.

Micelles formed by surfactants, lipids, or other amphiphilic molecules are the simplest building blocks of supramolecular assemblies. Above the critical micellar concentration (CMC), these molecules organize into spherical single- or double-layered assemblies, exposing the hydrophilic part of the molecules to the solvent and the hydrophobic groups toward each other through hydrophobic interactions. While amphiphilic molecules are long-known for their ability to suspend SWCNTs, they do not necessarily maintain their spherical micellar supramolecular shape on the surface of the SWCNTs,^50^ despite the naïve assumption that SWCNTs could have been easily integrated into micellar structures with the hydrophobic part of the amphiphile being oriented inward perpendicular to the SWCNT surface and the hydrophilic part facing outward to the solvent. Instead, the molecular self-assembly on the nanotube surface is more complex and is influenced by the molecular structure and concentration of the surfactants.^40,49,51−53^ For example, molecular dynamics simulations have suggested the lipid dipalmitoylphosphatidylcholine (DPPC) forms cylindrical micelles around SWCNTs, whereas dihexanoylphosphatidylcholine (DHPC) and lysophosphatidylcholine (LPC) form supramolecular assemblies of half-cylinders or hemimicelles on the nanotube surface (Figure 1a).^54^ Conversely, the surfactant sodium dodecylbenzenesulfonate (SDBS) results in disordered aggregates on the SWCNT surface.^51^ A recent study systematically investigating the morphology of the coatings of the surfactant sodium dodecyl sulfate (SDS) on SWCNTs across a wide range of SWCNT diameters and surfactant concentrations, revealed that at low SDS concentrations, the alkyl chains of the SDS molecules aligned orderly along the nanotube axis.^49^ As the SDS concentrations increased, the alignment became more random, and at the highest concentrations, micellar SDS clusters encapsulated the SWCNTs (Figure 1b). This behavior was depicted in a morphology phase diagram, demonstrating minimal influence of the SWCNT diameter on the morphology (Figure 1c).^49^ Given these complexities of micellar behavior, the presence of SWCNTs can significantly alter the structural integrity and morphology of these supramolecular assemblies, underscoring that the formation of self-assemblies by these molecules does not guarantee their typical structure when combined with SWCNTs.

In terms of applications, micellar assemblies facilitate the creation of nonpolar microenvironments around the SWCNTs, which was shown to be useful for studying the solvatochromic shifts in SWCNT fluorescence in response to varying solvent polarity, or the synthesis of ultrathin polymer-shells around the SWCNTs.^55−57^ Further, the molecular self-assembly, the micellar structure formed around the SWCNTs, and potential disassembly or degradation, inducing distinct dielectric environment changes around the SWCNTs, can be monitored through SWCNT spectroscopic signals, such as vibrational sum frequency generation^52,53^ and fluorescence measurements.^42^

Integrating SWCNTs into supramolecular assemblies provides a valuable method to investigate their composition and degradation, as the fluorescence emission of the SWCNTs is sensitive to changes in the dielectric environment induced by the surfactants or amphiphiles on the SWCNT surface. Wulf et al. investigated the enzymatic degradation of polymer dendron amphiphiles, which form micellar assemblies, and were shown capable of suspending SWCNTs.^42,58^ The polymer dendron amphiphiles consisted of a hydrophilic polyethylene glycol (PEG) chain linked to a hydrophobic dendritic branching unit with different hydrophobic end-groups that were coupled via ester bonds in different orientations (naphthyl, naphthoate, pentyl, hexanoate) (Figure 1d). These ester bonds are susceptible to enzymatic cleavage by esterase. The end-groups were shown to have different interactions with the SWCNT surface, manifested, on the one hand, in distinct fluorescence emission of the SWCNTs and, on the other hand, in their susceptibility to enzymatic degradation. The enzymatic degradation of the PEG-dendrons was followed by HPLC and absorption spectroscopy and was correlated to the SWCNT fluorescence signal monitored during the degradation process (Figure 1e). The HPLC data, measuring the cleavage of the free micelles, correlated with the fluorescence signal of the SWCNTs, proving that, in this case, the properties of the micellar structure around the SWCNTs and the free micelles are comparable. The PEG-dendrons with pentyl and hexanoate showed similar NIR fluorescence and similar reactivity to the enzyme, whereas naphthyl and naphthoate showed very different fluorescence spectra. This effect was attributed to the interaction of the end group with the SWCNT surface. While pentyl and hexanoate interacted through simple hydrophobic interactions, naphthyl and naphthoate could build π–π-interactions with the sp^2^-lattice of the SWCNTs. These π–π-interactions, in turn, were influenced by the different positions of the ester bond, which either introduces an electron donor or an electron acceptor in the direct vicinity of the aromatic group, affecting its electron density. Therefore, the PEG-dendrons with the naphthoate group showed minimal susceptibility to enzymatic degradation.^42^ This nuanced understanding of the interactions between the amphiphilic structures and SWCNTs not only illuminates the complex dynamics of supramolecular assemblies on the SWCNT surface but also underscores the potential for tailored functionalization strategies to enhance the functionality of SWCNT-based systems.

## 3 SWCNTs’ Interaction with Supramolecular Ring-Forming Structures

Apart from micellar assemblies, there are supramolecular complexes, designed from synthetic molecules or biomolecules that form precisely defined ring structures. If these complexes possess appropriate geometries, such as suitable inner ring diameter and molecular composition, they can arrange around the SWCNTs and stabilize them in suspension.^59,60^ Due to their defined size, i.e., inner diameter, many ring-structured molecules are chirality-selective when suspending SWCNTs.

**Figure 2:**
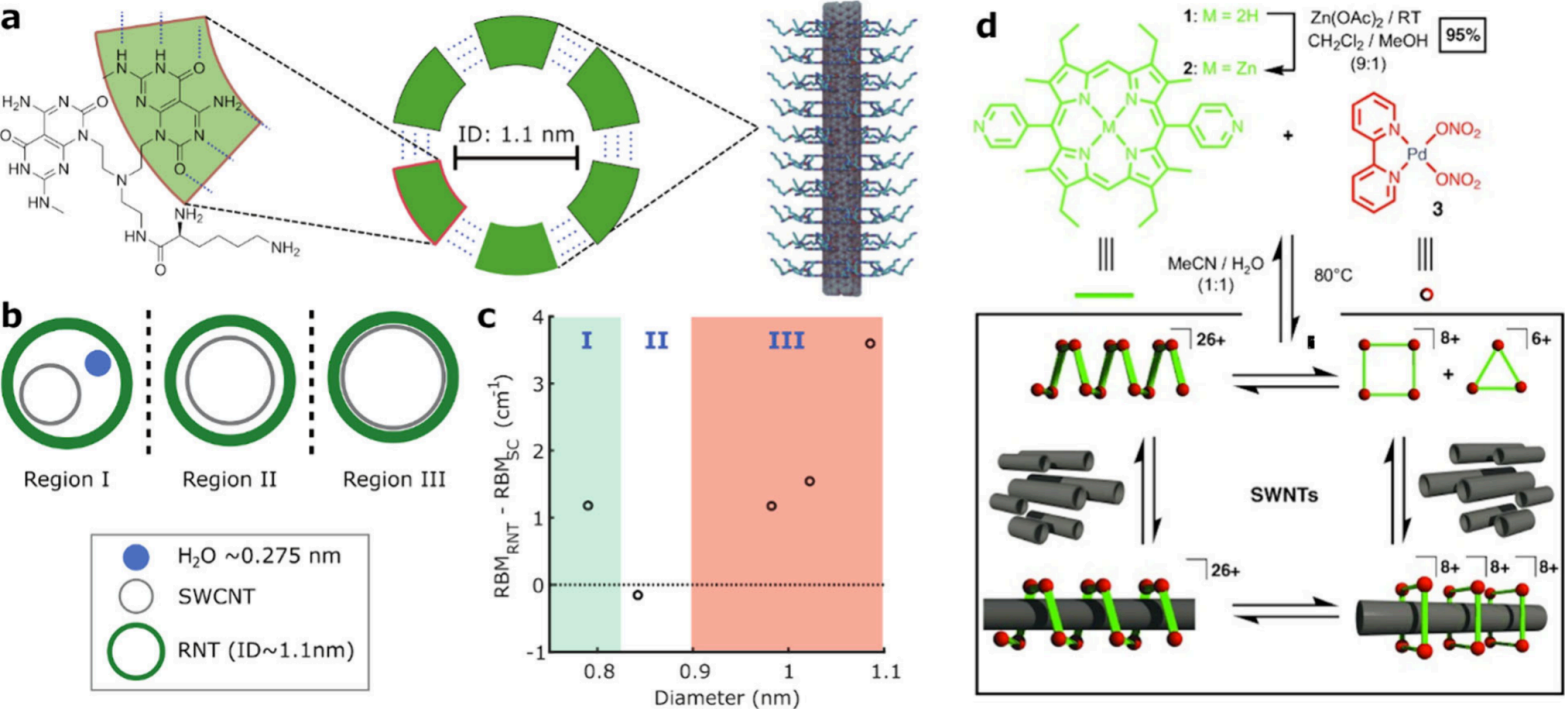
SWCNTs suspended with supramolecular ring structures. a) The heteroaromatic bicyclic bases self-assembled into six-membered rings with an inner diameter of 1.1 nm. The rosette ring structures stack to rosette nanotubes (RNT) and incorporate SWCNTs. b) Different configurations of RNT encapsulating SWCNTs, depending on the SWCNT chirality. For small SWCNT diameters, water molecules can be present in the space between the RNT and SWCNT (Region I); at intermediate diameters, water is excluded, and only minimal space is left between the RNT and SWCNT (Region II); at larger diameter, SWCNTs are in direct contact with the RNTs (Region III). c) Raman-signal of SWCNT-RNTs compared to sodium cholate suspended SWCNTs, as a function of the SWCNT diameter reflecting the three regions. Reprinted with permission from ref (60). Copyright 2022 Wiley. d) Supramolecular complexes built from porphyrins and a cis-protected Pd^II^-complex from either ring structures or acyclic oligomers, resulting in a helical structure around the SWCNTs, depending on their diameter. Reprinted with permission from ref (59). Copyright 2005 Wiley.

Rosette nanotubes (RNT) exemplify this, as they are formed by the supramolecular self-assembly of heteroaromatic bicyclic bases into six-membered rings held together by hydrogen bonds. These rosettes stack into channels extending several micrometers in length.^60,61^ Gong et al. demonstrated that these RNTs could serve as a precise corona phase to stabilize SWCNTs in water. Notably, functional groups covalently attached to the outside of the rosette subunits could modify the SWCNT surface and tailor their properties and dimensions (Figure 2a). The RNTs formed a tightly packed wrapping around SWCNTs, leaving only minimal exposed surface area. Measuring the suspension yield via the photoluminescence of the SWCNT-RNTs showed a selective preference of RNTs toward SWCNTs with a larger diameter. In particular, a significant photoluminescence increase of 263-fold of the (11,1) chirality was observed and attributed to an optimal electron density offset between this chirality and the RNT. Further analysis via Raman spectroscopy identified three distinct local environments for the SWCNTs within the RNTs (Figure 2b): I) A high solvent exposure for small diameter SWCNTs in the RNTs; II) An exclusion of water with some spacing left between the SWCNT and the RNT; III) An increased interactions between the SWCNTs and the RNTs for larger diameters, evident by a red shift of the SWCNT-RNT Raman signal compared to SWCNTs suspended by sodium cholate (Figure 2c).^60^

Chichak et al. developed a supramolecular complex composed of free-base or Zn^II^ porphyrin and a cis-protected Pd^II^-complex, which, under thermodynamic control, could form either cyclic structures or acyclic oligomers (Figure 2d). An equimolar mixture of the Zn^II^ porphyrin and the Pd^II^-complex was shown capable of suspending SWCNTs, where molecular dynamics simulations indicated that the ability of the complex to form cyclic and acyclic forms around the SWCNTs depended on the SWCNT diameter.^59^ This adaptability allowed for the tailored conformation of the complex according to the specific dimensions of the SWCNTs, providing a versatile means of suspension. A similar square-shaped supramolecular organometallic complex based on N-heterocyclic-carbenes instead of porphyrenes and joined by palladium-complexes was reported to form mechanically interlocked carbon nanotubes (MINTs).^62^ MINTs are considered as rotaxane-like species with the SWCNT as a thread.^63^ These suspensions show high stability, while preserving the SWCNT structure and find application as polymer reinforcement,^64^ catalysts,^65^ or qubits.^66^

The interaction of SWCNTs with supramolecular structures that form ring-like configurations showcases the potential of molecular design in stabilizing SWCNTs through strategic molecular encapsulation. In particular, the formation of ring structures with defined diameters can advance research on chirality selective suspension of SWCNTs. The ability of these supramolecular assemblies to adapt their form or to match the dimension of the SWCNTs highlights their potential for creating highly specialized coronae for advancements in the development of nanomaterials.

## 4 SWCNTs and Supramolecular Assemblies of DNA (DNA-Origami)

The base-paring of DNA strands enables them to hybridize and, with proper sequencing, to fold into highly specific supramolecular 2D or 3D structures. This principle is utilized in DNA-origami techniques, where long single-stranded DNA with a precisely defined base sequence, is folded into a predetermined structure, with the support of short oligonucleotide staple strands.^67−69^ Many studies present sophisticated functional DNA-origami structures, such as DNA machines,^70^ with emerging potential for practical applications. Due to the nanometer-resolution manufacturing of 2D or 3D structures of DNA-origami, these materials have been applied for the precise positioning of nanoparticles, facilitating, for example, the formation of chiral plasmonic structures of gold nanorods.^71,72^

**Figure 3:**
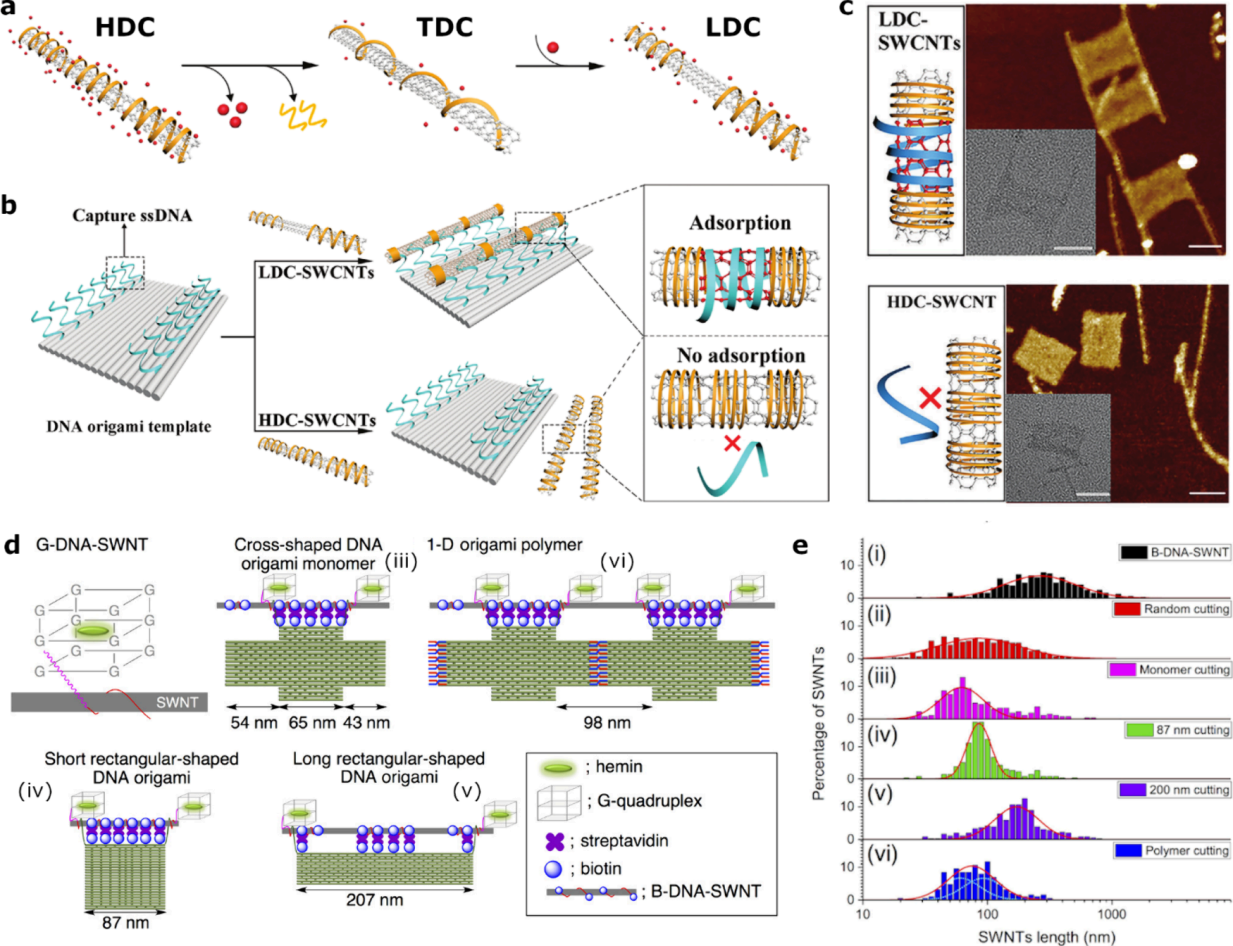
SWCNTs integrated in DNA-origami. a) To induce ionic-strength-mediated corona defects, SWCNTs are initially suspended by DNA in a high NaCl concentration solution to achieve a high-density corona phase (HDC). When the NaCl concentration is lowered, all excess DNA detaches and is removed to form a transition DNA corona phase (TDC). Increasing the NaCl concentration again results in a low-density corona phase (LDC), leaving a defective corona phase. b) A DNA-origami template with capture DNA strands enables precise and efficient positioning of LDC-SWCNTs, whereas HDC-SWCNTs do not adhere to the template. c) AFM images show parallel assembly of SWCNTs on the DNA-origami templates. Reprinted with permission from ref (79). Copyright 2024 Wiley. d) DNA origami templates designed in various shapes that expose G-quadruplex/hemin complexes in defined distances, facilitating the defined rupture of SWCNTs bound to these DNA-origami templates. e) Measured length distributions for the (i) as-suspended SWCNTs, (ii) randomly cut with free G-quadruplex/hemin complexes in solution, and (iii–vi) the different templates shown in d). Reprinted with permission from ref (82). Copyright 2018 American Chemical Society.

SWCNTs vary in electronic properties, being metallic or semiconducting, based on their structure. These properties render them useful for electronic nanodevices, given the exactly defined positioning of the nanotubes.^73^ Single-stranded DNA (ssDNA) is a versatile suspending agent of SWCNTs, especially for their use as biosensors for imaging and fluorescence sensing, due to the biocompatibility and the variability of DNA sequences.^74^ Further, ssDNA has proven to show chirality selective affinity to SWCNTs.^75,76^ These affinities between SWCNTs and DNA enables us to integrate them into DNA-origami platforms and it can facilitate molecular-level design. Using DNA-origami templates, precise positioning of SWCNT has been achieved in various configurations, including angular,^77−79^ parallel,^79,80^ or triangular.^81^ In a recent study, Luo et al. enhanced the binding efficiency of SWCNTs to DNA-origami surfaces and improved their positioning accuracy using ionic-strength-mediated DNA corona defects (DCD).^79^ The SWCNTs were tethered to the DNA-origami template via capture DNA strands that exhibit a strong affinity to the SWCNT surface, effectively wrapping around them. As the SWCNTs were also suspended by a DNA corona phase, the capture strands could bind more efficiently when the DNA corona is of low density, i.e., containing DCDs (Figure 3a). This low-density phase was achieved by initially suspending the SWCNTs with DNA in a high NaCl concentration, promoting a high-density corona phase (HDC) of DNA due to enhanced self-stacking of nucleobases. Subsequently, the salt concentration was substantially lowered, which decreased the coverage by allowing excess DNA to desorb, forming a transition DNA corona phase (TDC). A subsequent increase in salt concentration locally compacted the DNA conformation into low-density corona phase (LDC), leaving gaps of accessible surface area on the SWCNT surface. These gaps acted as anchoring points to the DNA origami template, enhancing the efficiency of attachment to the templates at low salt concentrations (Figure 3b). The specific design of the DNA origami templates facilitated the efficient arrangement of SWCNTs in a parallel confirmation (Figure 3c), optimizing their functional integration into the designed nanostructures.^79^

Apart from serving as templates for precise SWCNT positioning, DNA-origami assemblies have also facilitated the precise cutting of SWCNTs into defined lengths,^82^ addressing the issue of high length-polydispersity of synthesized SWCNTs. To achieve a narrower length distribution, Atsumi et al. developed a DNA-origami platform incorporating G-quadruplex DNA sequences, which form cage-like structures capable of complexing hemin. Hemin catalytically oxidizes H_2_O_2_ into radical oxygen intermediate products that can induce structural defects in the SWCNTs, ultimately rupturing them. This approach allows for cutting SWCNTs at predefined lengths using various geometries of DNA-origami platforms, where the position of the G-quadruplex/hemin complexes determines the cutting sites of the SWCNTs that are attached to the origami templates via streptavidin/biotin binding. These structures contain cross-shaped DNA origami monomers that can be assembled to an origami polymer, and two rectangular structures of 87 and 207 nm cutting lengths (Figure 3d). Atomic force microscopy (AFM) measurements confirmed that the length distributions of the resulting SWCNT pieces corresponded closely to the specifications of the DNA-origami templates used for the cutting (Figure 3e).

This exploration into the interplay between SWCNTs and supramolecular DNA-origami templates demonstrates the profound potential of DNA-based nanostructures in advancing nanotechnology applications. By leveraging the precision and versatility of DNA-origami, SWCNTs can be manipulated with high accuracy, achieving precise positioning and cutting, thus enabling numerous future applications, such as electronic devices with aligned SWCNTs.^83,84^

## 5 Self-Assembling Amino Acids, Peptides, and Proteins Forming Stable SWCNT-Suspensions

Aromatic amino acids, peptides, and also several proteins have the capability to form stable self-assemblies that can organize into defined 3D structures, linear fibers or tubes, or even hydrogels.^33,85−87^ Although their interaction with SWCNTs is less explored than DNA-based approaches, the array of amino acid building blocks is chemically more diverse as peptide chains are a versatile group of macromolecules, giving rise to extensive variability in structure and function. Certain amino acids, peptides, and proteins can form supramolecular self-assembled structures that are able to effectively suspend SWCNTs in solution or integrate suspended SWCNTs within these assemblies.

SWCNTs can be suspended by amino acid or peptide derivatives like naphthalene or fluorenylmethoxycarbonyl (Fmoc) derivatives, both of which are known to form supramolecular self-assemblies.^12,43,44,88,89^ Similar to micellar assemblies, it has not yet been demonstrated whether the predominantly fibrous structure of these self-assembled amino acids is maintained when integrated with SWCNTs.^43^ Notably, in a multicomponent system based on the self-assembly and subsequent oxidative enzymatic polymerization of Fmoc-tyrosine suspended SWCNTs, used as a metal sensor system, Wulf et al. showed variations in the morphology of the Fmoc-tyrosine fibers in the presence of SWCNTs,^43^ while SWCNTs integrated into Fmoc-diphenylalanine hydrogels did not show this effect.^12^ Nevertheless, further research on the integration of SWCNTs into self-assembled Fmoc-amino acid fibers is of interest.

**Figure 4:**
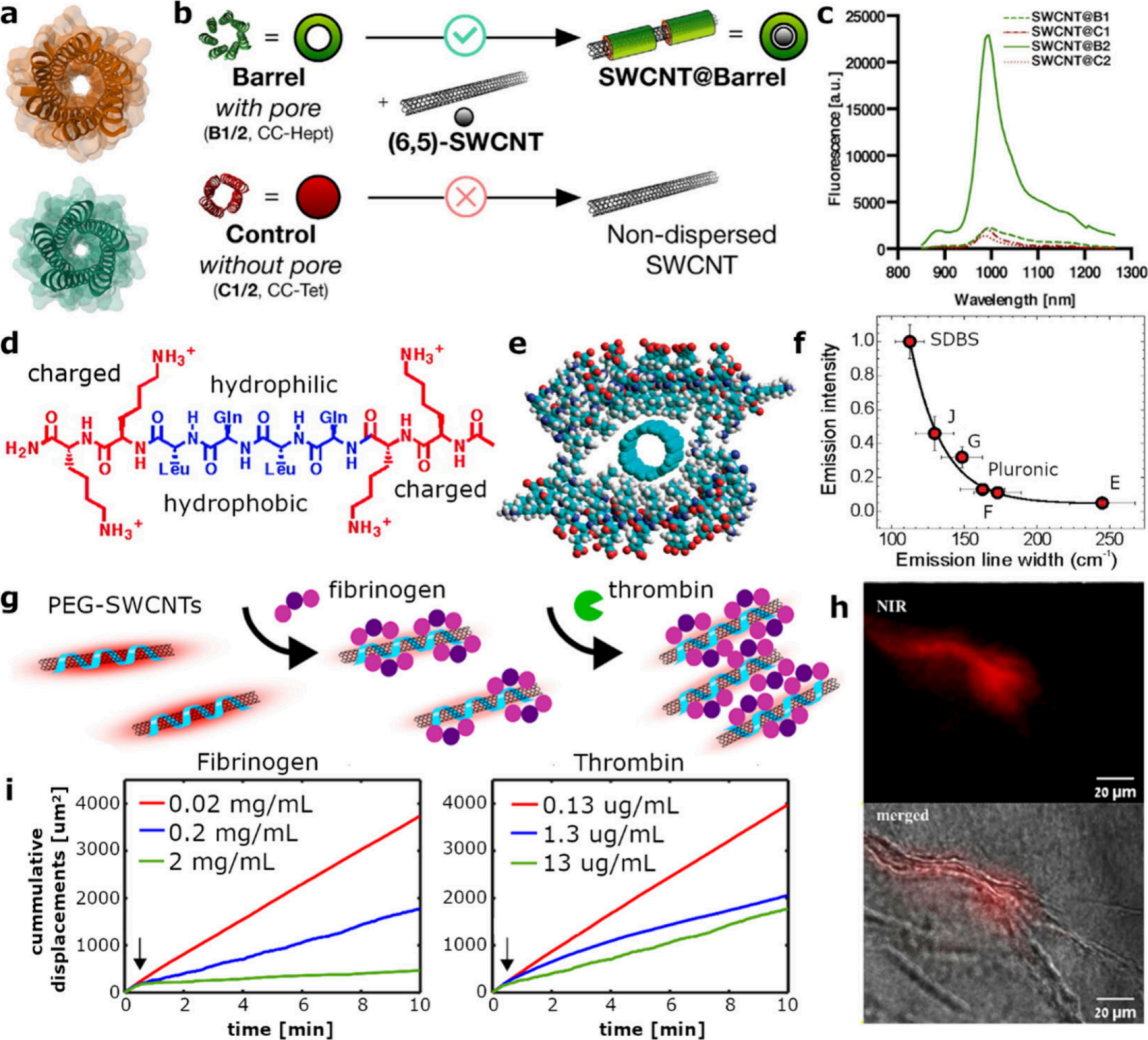
SWCNTs suspended by peptide or protein supramolecular assemblies. a) The structure of two barrel-forming coiled-coil peptides, PDB ID 4pn8 (CC-Pent, green) and 4pn9 (CC-Hex2, orange) displaying the different diameters of the cavity. b) Two different barrels used for suspending SWCNTs, CC-Hept (B1/B2) with a pore size of 0.76 nm that can suspend SWCNTs and CC-Tet (C1/C2) with a small pore size of 0.39 nm that cannot suspend SWCNTs. c) Fluorescence spectra of the various SWCNT suspensions showing that modified peptide B2 is most efficient in suspending SWCNT. Reprinted with permission from ref (92). Copyright 2018 Wiley. d) Multidomain peptide structure with ABA block motif, where A is the charged lysine sequence and B is the altering glutamine and leucine sequence, which arrange to form a hydrophilic and a hydrophobic side, respectively. Reprinted with permission from ref (95). Copyright 2007 American Chemical Society. e) Self-assembled ABA multidomain peptides suspending a SWCNT in their hydrophobic cavity. f) Photoluminescence emission intensity as a function of the emission line width for different peptide sequences (E, F, G, and J) compared to SDBS and Pluronic F-127 suspended SWCNTs. Reprinted with permission from ref (91). Copyright 2008 American Chemical Society. g) Thrombin-mediated self-assembly of fibrinogen incorporating SWCNTs. Fibrinogen initially binds to PEG-functionalized SWCNTs. After the addition of thrombin, fibrinogen is converted to insoluble fibrin and self-assembles into fibers on the SWCNT surface. h) NIR fluorescence and merged NIR and brightfield image of fibrin fibers and the NIR fluorescence signal of the integrated SWCNTs. (i) Time-dependent cumulative displacement of SWCNTs before and after (arrow) the addition of thrombin. Left: fibrinogen concentration of 0.02 mg mL^–1^ (red), 0.2 mg mL^–1^ (blue), and 2 mg mL^–1^ (green) upon the addition of 13 μg mL^–1^ thrombin. Right: fibrinogen concentration of 0.02 mg mL^–1^ upon the addition of thrombin at 0.13 μg mL^–1^ (red), 1.3 μg mL^–1^ (blue), and 13 μg mL^–1^ (green). Reprinted with permission from ref (98). Copyright 2023 American Chemical Society.

Peptide self-assemblies have been explored for suspending SWCNTs using barrel-like structures formed from coiled-coil assemblies of helices,^90−92^ which vary in size and inner diameter based on the number of peptide chains (Figure 4a).^93,94^ The efficiency of SWCNT encapsulation by these coiled-coil peptide barrels was shown to be dependent on the size of their inner cavity.^92^ For example, the coiled-coil heptamer barrel (CC-Hept), with an inner cavity of 0.76 nm, could suspend (6,5)-SWCNTs, whereas a coiled-coil tetramer barrel (CC-Tet) with a much smaller inner cavity of only 0.39 nm could not (Figure 4b and c). Further refinement of this selective suspension was achieved by modifications in the amino acid sequence of the peptides. In another approach, multidomain peptides organized in an ABA block motif have shown promise in suspending SWCNTs.^91,95^ The A-domain consisted of charged amino acids (lysine, K), while the B-domain consisted of altering hydrophobic (leucine, L) and hydrophilic (glutamine, Q) residues. These peptides fold in β-sheet structures with charged ends, with distinct hydrophobic and hydrophilic sides (Figure 4d). Peptides of different domain lengths were tested for their ability to suspend the SWCNTs, and the photoluminescence intensity of single SWCNTs obtained from fluorescence imaging was compared to that of SWCNTs suspended by sodium dodecylbenzenesulfonate (SDBS), which is a bioincompatible surfactant yet giving rise to high SWCNT photoluminescence. The study identified three peptides that formed β-sheet structures, as confirmed by CD-spectroscopy, and successfully suspended SWCNTs (Figure 4e), namely, peptide E (K_3_(QL)_6_K_3_), peptide F (K_2_(QL)_6_K_2_), and peptide G (K_3_(QL)_5_K_3_). Further, peptide G, which resulted in the highest photoluminescence intensity, was modified with phenylalanine instead of the leucine as a hydrophobic side chain (Peptide J), achieving 40% of photoluminescence intensity of the SWCNT-SDBS (Figure 4f).^91^

In a further study, SWCNTs suspended with two multidomain peptides of similar structure as described above were subjected to mouse fibroblasts. The *in vitro* cytotoxicity of the SWCNT-peptide composites was tested and compared to SWCNT samples suspended with SDBS and two surfactants from the Pluronic series. The peptide-functionalized SWCNTs showed dose-dependent cytotoxicity, but were not significantly different from the Pluronic-SWCNTs, while SDBS-functionalized SWCNTs were shown to be highly cytotoxic.^96^

Additionally, the self-assembled tubular structure of the tobacco mosaic virus (TMV) protein coat has also been demonstrated to maintain SWCNTs in suspension.^97^ The protein coat of TMV is a 300 nm long cylinder of 18 nm in diameter and could be functionalized on its exterior with a defined number of ketone groups. SWCNTs were functionalized with ketone reactive pyrene groups, that could be coupled to the protein coat of TMV.^97^ These studies highlight the potential for embedding SWCNTs within predefined peptide supramolecular self-assembly structures, enabling the exploration of further practical applications.

Gerstman et al. presented a SWCNT sensor for blood coagulation based on monitoring the self-assembly of fibrin on the SWCNT surface, which is formed by the conversion of fibrinogen to an insoluble form triggered by the serine protease activity of thrombin.^98^ The sensor was based on fibrinogen absorbed on PEG-functionalized SWCNTs in a fibrinogen solution.^99^ Upon the addition of thrombin, fibrinogen was cleaved to fibrin monomers, which self-assembled into fibrin fibers incorporating the SWCNTs (Figure 4g), as confirmed by the colocalization of the NIR SWCNT fluorescence and the fibrin fibers (Figure 4h). Further experiments measured the cumulative displacement of the SWCNT particles before and after the addition of thrombin, revealing a reduction in the diffusivity of the SWCNTs over time with a decrease rate depending on both the fibrinogen concentration and the concentration of thrombin (Figure 4i). This slowdown in diffusivity was attributed to the formation of fibrin clots, which depended on the concentration of both reactants, thus serving as a proxy for the amount of clottable fibrinogen and active thrombin. Consequently, the SWCNTs were effectively utilized as a NIR optical sensor for monitoring the thrombin-mediated coagulation behavior of fibrinogen.^98^

These demonstrations of the self-assembly of amino acids, peptides, and proteins into supramolecular structures incorporating SWCNTs underscore the transformative potential of these nanoparticle-biomolecule hybrids for advanced biomedical applications and sensor technologies.

## 6 SWCNTs in Hydrogels Formed by Self-Assembly of Low Molecular Weight Gelators

Low molecular weight gelators (LMWGs) are small organic molecules that, at certain concentrations, can self-assemble into supramolecular self-supported hydrogels with fibrillary networks. Similar to surfactants in their amphiphilicity and limited water solubility, LMWGs differ in their tendency to form fibrillary or tubular backbones rather than spherical micelles, due to their molecular structure.^100^ Typically, LMWGs are homogeneously dissolved in a specific solvent, pH or temperature, such that changing these conditions (solvent polarizability, pH change, temperature change) increases the molecules’ affinity to each other, leading to their self-assembly into fibrillary structures that entangle to form hydrogels. Most prominent examples of LMWGs are amino acids and peptides,^85^ along with their derivatives and conjugates, yet other biomolecules like bile acid,^101,102^ saccharide derivatives, or entirely synthetic organic molecules also show self-assembly capabilities. Often, the properties of LMWG-based hydrogels are enhanced by additives such as polymers or nanomaterials like SWCNTs,^32^ particularly valuable in biomedical engineering applications, where these hydrogels serve as platforms for tissue engineering, wound healing, or injectable and implantable hydrogels.^103−105^

SWCNTs, with their inherent flexibility and tubular structure, are predestined for integration into the fibrillary network of hydrogels. In many studies, SWCNTs, in their oxidized, water-soluble form, are integrated into LMWG-hydrogels, primarily serving as structural supporting agents for the hydrogel network.^14,33,106,107^ Nevertheless, when used as pristine SWCNTs, maintaining their optical and electronic characteristics, few studies have leveraged these properties to enhance the functionality of the hydrogels beyond merely stabilizing the hydrogel network. This utilization suggests significant potential for innovative applications that capitalize on the distinctive properties of SWCNTs to add value to supramolecular hydrogel systems.

The first stable dispersion of pristine SWCNTs in a LMWG-hydrogel was achieved using a monosaccharide-azonaphthol conjugate (β-d-glucopyranoside-azonaphthol), where the incorporation of SWCNTs lead to an increased sol–gel transition temperature of 10 °C.^108^ A SWCNT concentration-dependent increase of the storage modulus of l-phenylalanine derivative hydrogels was observed when the N-terminus of the amino acid was conjugated to various hydrophilic groups (C-16, naphthalene, pyrene) and the C-terminus was conjugated to triethylene glycol.^109^ Additionally, an artificial amino acid–based hydrogel containing SWCNTs as a structural additive showed a higher storage and loss modulus when containing SWCNTs. Further, the self-recovery time to a hydrogel after applying mechanical stress leading to gel–sol transition was reduced from 7 min for a native gel to 2.5 min for a gel containing SWCNTs.^110^ In another approach, a supramolecular hydrogel based on guanosine complexation with K^+^ ions was also found to benefit from the incorporation of SWCNTs. The gels showed a SWCNT-concentration-dependent increase in storage and loss moduli as well as shear stress tolerance. The elastic recovery for a step-strain experiment increased to 100% with the highest SWCNT concentration compared to 84% without SWCNTs. The resulting gels were shown to be ideal supporting materials for cellular growth.^111^

**Figure 5:**
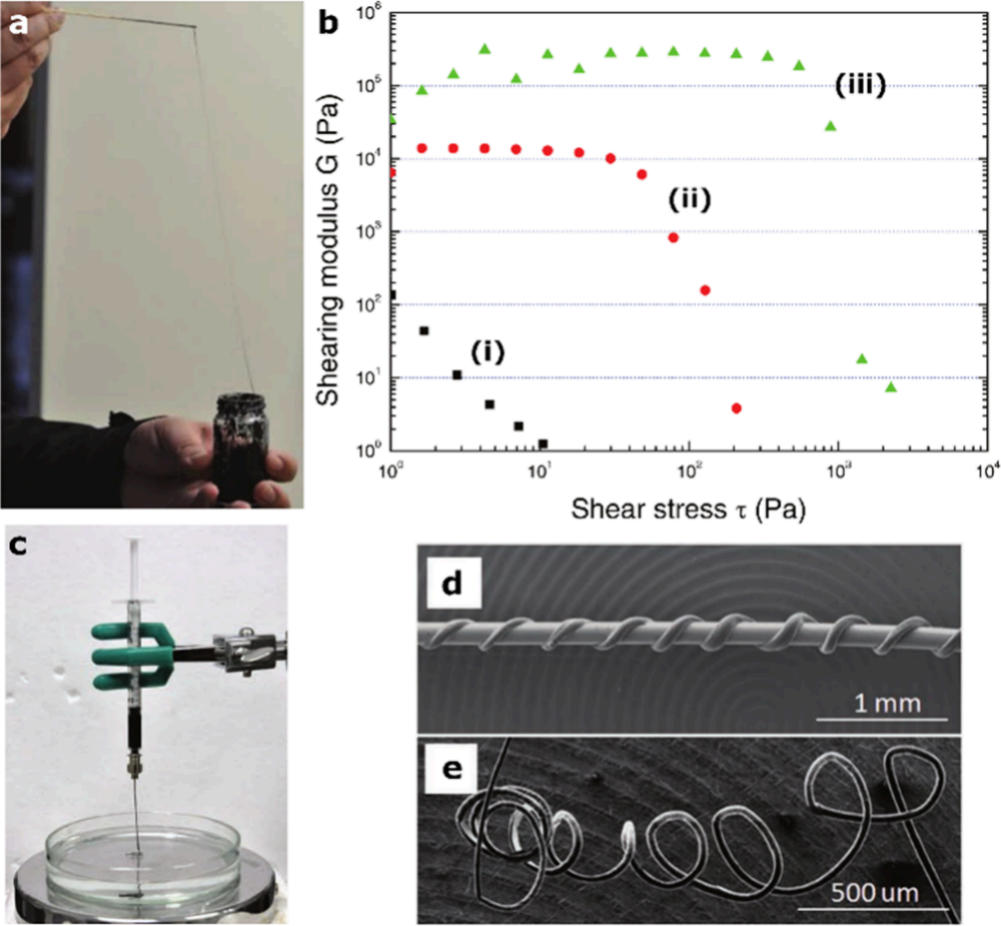
SWCNTs integrated into hydrogels formed by bile salt. a) Stretching capacity of the hydrogel. b) Shear modulus, *G*, of NaDC hydrogels with SWCNTs in different concentrations as a function of the shear stress *t*, (i) 1% SWCNTs, (ii) 2% SWCNTs, (iii) 3% SWCNTs. c) Experimental setup used for producing printed hydrogel structures with a solvent-exchange process in ethanol. d) SEM image of a copper wire with a spiral nanopattern of the SWCNTs in the hydrogel. e) Free-standing spiral nanopattern. Reprinted with permission from ref (101). Copyright 2011 Wiley.

Leveraging the electron conductivity of SWCNTs, Tan et al. developed a hydrogel consisting of a bile salt, sodium deoxycholate (NaDC). Hydrogelation was initiated by adding SWCNTs to a highly concentrated NaDC solution. The resulting hydrogels exhibited elastic properties of extending up to 50-fold along the stretching direction (Figure 5a). Moreover, the shear modulus of the hydrogels increased significantly with the concentration of SWCNTs (Figure 5b). The material was utilized as “solid ink” to micropattern electronic devices through a solvent exchange process, where it was extruded through a micronozzle into ethanol solution (Figure 5c). In the ethanol environment, the NaDC molecules dissolved, whereas the SWCNTs concentrated in the printed micropattern through van-der Waals interactions. These printed structures, including wires and freestanding spirals with diameters as small as 30 μm (Figure 5d and e), revealed improved conductivity with a linear ohmic response in the I–V measurements compared to the SWCNTs in an intact hydrogel.^101^ In another example demonstrating the functionality of SWCNT additives, hydrogels consisting of a C_16_ amine derivative of l-alanine (16-A) and the redox-active viologen (V^2+^) were examined. Interestingly, the hydrogels lacking SWCNTs displayed only poor reversibility of the two-electron reduction of viologen. However, the incorporation of SWCNTs led to a quasi-reversible reduction with potentially switchable oxidation states, highlighting their role in enhancing the electrochemical properties of the hydrogel.^112^

**Figure 6:**
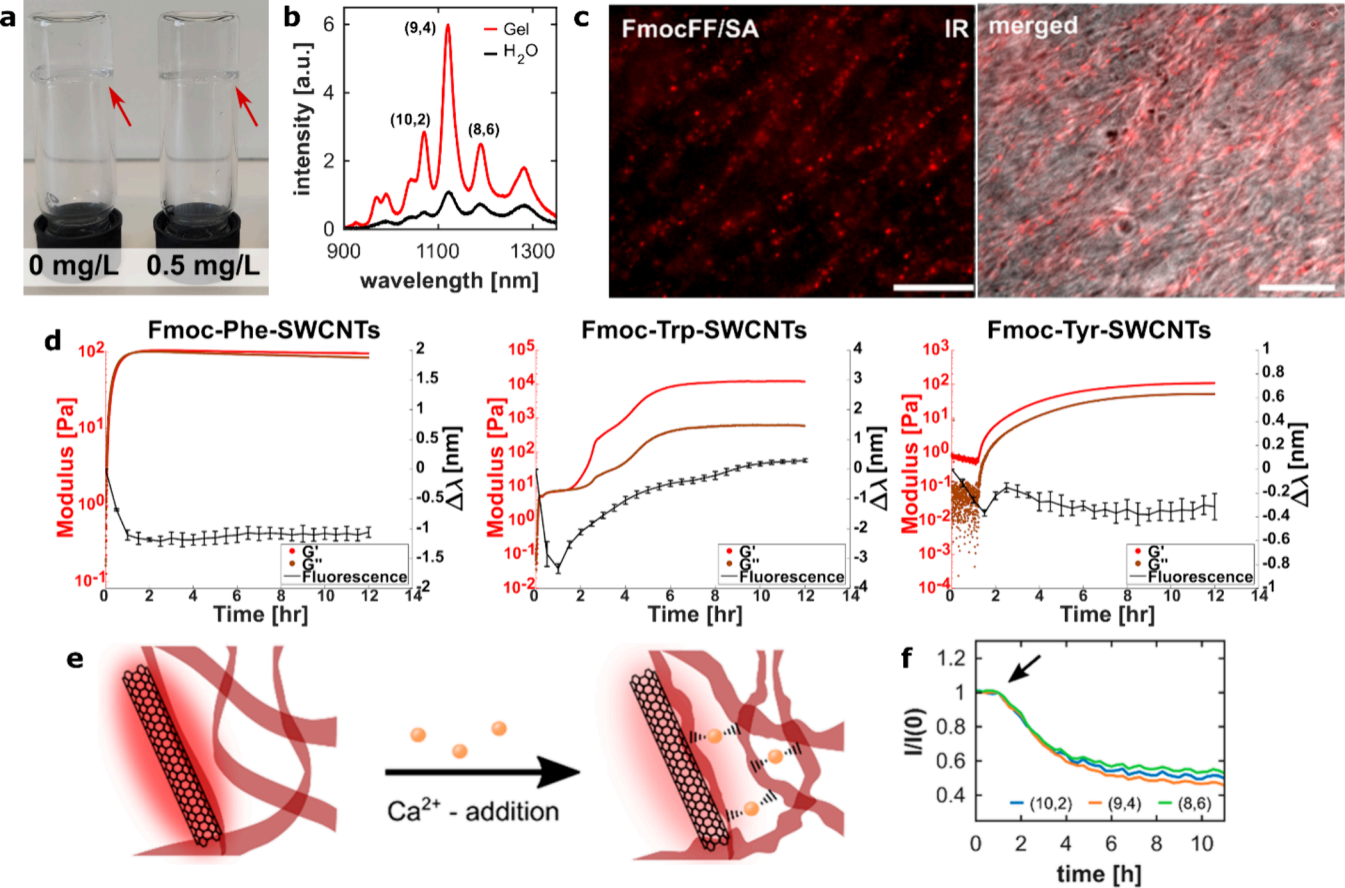
SWCNTs integrated into Fmoc-amino acid or Fmoc-peptide hydrogel as optical sensors. a) Integration of SWCNTs at low concentrations keeps the hydrogels transparent. b) Fluorescence signal of SWCNTs in Fmoc-phenylalanine (FmocFF) hydrogels. c) NIR fluorescence and merged NIR fluorescence and brightfield image of SWCNTs in FmocFF/sodium alginate (SA) hybrid hydrogels. Reprinted with permission from ref (12). Copyright 2022 American Chemical Society. d) Correlation between changes in mechanical properties (red: storage modulus *G*′; brown: loss modulus *G*″) of Fmoc-amino acid hydrogels and the fluorescence signal (black) of integrated SWNTs during the gelation process for Fmoc-phenylalanine (Fmoc-Phe), Fmoc-tryptophan (Fmoc-Trp), and Fmoc-tyrosine (Fmoc-Tyr). Reprinted with permission from ref (113) Copyright 2024 Wiley. e) Addition of Ca^2+^-ions to FmocFF/polymer hydrogels can lead to additional cross-linking of the hydrogels. f) Fluorescence changes of the SWCNTs in a FmocFF/SA hybrid hydrogel upon the addition of Ca^2+^-ions (arrow), leading to cross-linking between SA-polymers. Reprinted with permission from ref (12). Copyright 2022 American Chemical Society.

In all the above examples, SWCNTs were typically added in relatively high concentrations, resulting in opaque, black hydrogels. Under these conditions, the inherent optical properties of the SWCNTs, such as absorption and fluorescence, can hardly be effectively exploited. In a recent effort, SWCNTs were integrated as optical probes into hydrogels composed of Fmoc-amino acid (Fmoc-tryptophan, Fmoc-tyrosine, Fmoc-phenylalanine) and Fmoc-diphenylalanine/polymer hybrid hydrogels with additives of PEG, alginate, and dextran.^12,113^ These aromatic Fmoc-amino acids and Fmoc-peptides not only served as efficient dispersants for SWCNTs but also facilitated their self-assembly into hydrogels. By adding SWNCTs at low concentrations, ranging from 0.5 to 5 μg mL^–1^, the hydrogels remained transparent, and the SWCNTs retained their fluorescence properties within the hydrogel matrices (Figure 6a and b). Rheological measurements and electron microscopy confirmed that these low concentrations of SWCNTs did not alter the structural or mechanical properties of the hydrogels. Importantly, fluorescence imaging of the SWCNTs integrated within the hydrogels showed that they could reveal structural properties of the hydrogels and serve as an NIR-staining for hydrogels (Figure 6c). Given that peptide hydrogels are often used as injectable materials, a staining agent detectable in the NIR transparency window of biological tissue which does not suffer from photobleaching, offers significant advantages. Additionally, the gelation process of the hydrogel could be monitored through time-dependent fluorescence-spectroscopy of the integrated SWCNTs. The detected fluorescence wavelength changes during gelation time showed a correlation with the changes in mechanical properties measured via rheology (Figure 6d). Structural changes inside the formed hydrogels induced via the addition of a trigger such as Ca^2+^-ions, which acted as cross-linkers,^12^ or NaOH, which could lead to the dissolution of the hydrogels,^113^ were also tracked through the modulations in the SWCNTs fluorescence emission (Figure 6e and f).

## 7 Conclusion and Outlook

In this perspective, we have demonstrated that supramolecular assemblies offer considerable promise for the suspension or integration of SWCNTs, exploiting the properties of both components for enhanced functionality. Supramolecular assemblies with predefined structures, such as coiled-coil peptide barrels or rosette nanotubes, have been shown to selectively suspend specific SWCNT chiralities,^60,92^ and DNA-origamis have facilitated the precise positioning of SWCNTs for use in electronic nanodevices.^79,80^ Nevertheless, the full structural implications of integrating SWCNTs into various other supramolecular assemblies, like micelles formed by surfactants or lipids, and fibers formed by amino acids or peptides, depend on the assembling agents and remain to be further explored.

Furthermore, SWCNTs have demonstrated significant potential as optical sensors capable of monitoring the self-assembly and disassembly of supramolecular materials, like micellar structures, fibers, or hydrogels.^12,42,98^ While often incorporated in high concentrations to serve as stabilizing agents within supramolecular hydrogels, leading to a loss of optical transparency, at lower concentrations, the hydrogel remains transparent, and the SWCNTs can effectively report on the long-term state of the hydrogel. This current trade-off between stabilization and sensor functionality presents a challenge that invites further exploration into the nuanced interactions between SWCNTs and hydrogel components. By enhancing the functionalization strategies of SWCNTs and fine-tuning the molecular composition of hydrogels, it may be possible to optimize both stabilization and sensing capabilities without compromising the properties of both.

The integration of SWCNTs into these complex systems not only expands the utility of supramolecular assemblies but also allows for applications in areas such as biomedical engineering, electronic devices, and environmental sensing. Supramolecular assemblies enable the integration of SWCNTs into larger functional structures, where molecular-scale interactions with these nanoscale carbon nanotubes affect mesoscale properties, leveraging the characteristics of SWCNTs. This approach aims to create structures with emergent properties applicable across these scales. Further, recent efforts in covalently modifying SWCNTs with quantum defects that maintain and extend their optical properties could allow the integration of SWCNTs in a more controlled way to supramolecular structures. Given the growing interest in employing SWCNTs as NIR fluorescent optical sensors integrated within supramolecular assemblies, it is crucial to develop a deeper understanding of the interaction between SWCNTs with different supramolecular frameworks in future research to fully harness their potential in advanced material applications.

## Vocabulary:

Single-Walled Carbon Nanotubes: Carbon nanomaterials with a graphene lattice structure rolled into a cylinder, providing high electron conductivity, mechanical stability, and near-infrared fluorescence.
Supramolecular Assembly: A three-dimensional molecular structure consisting of molecules self-assembled via noncovalent bonds, e.g., van der Waals interactions, electrostatic interactions, and hydrogen bonds.
DNA-Origami: Three-dimensional structures assembled via controlled hybridization of designed DNA-strands. The design of the DNA sequence allows for the formation of structures with nanometer precision and the development of molecular machines.
Low Molecular Weight Gelators: Small organic molecules whose supramolecular self-assemblies form three-dimensional hydrogel networks.
Peptide Hydrogels: Hydrogels formed via the supramolecular assemblies of short peptides, often consisting of amino acids with high aromaticity.
